# Supplementary material for: Comparison of the Immunogenicity and Efficacy of rBCG-EPCP009, BCG Prime-EPCP009 Booster, and EPCP009 Protein Regimens as Tuberculosis Vaccine Candidates
Source: Vaccines (Basel). 2023 Nov 22;11(12):1738. doi: 10.3390/vaccines11121738 (PMC10747267; doi:10.3390/vaccines11121738)
Supplement: Supplementary file 1 [file vaccines-11-01738-s001.zip › vaccines-2651444-supplementary.pdf]

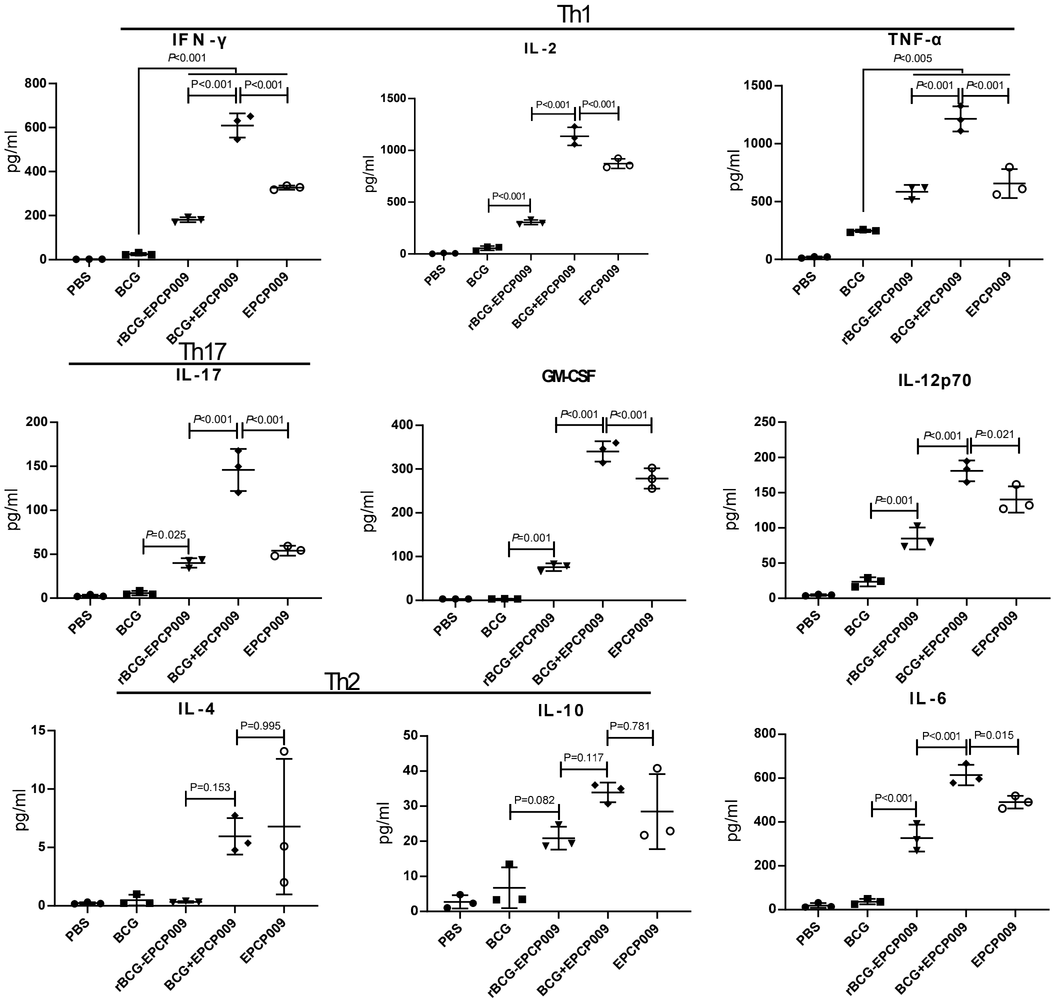

Figure S1. Levels of cytokines secreted by mouse spleen lymphocytes 8 weeks after immunization with the candidate vaccines. Cytokine levels were examined after stimulation with EPCP009 protein after 8 weeks of initial immunization with the different vaccine candidates. The p values indicating the significance of differences were determined by one-way ANOVA with Tukey's multiple comparison tests.

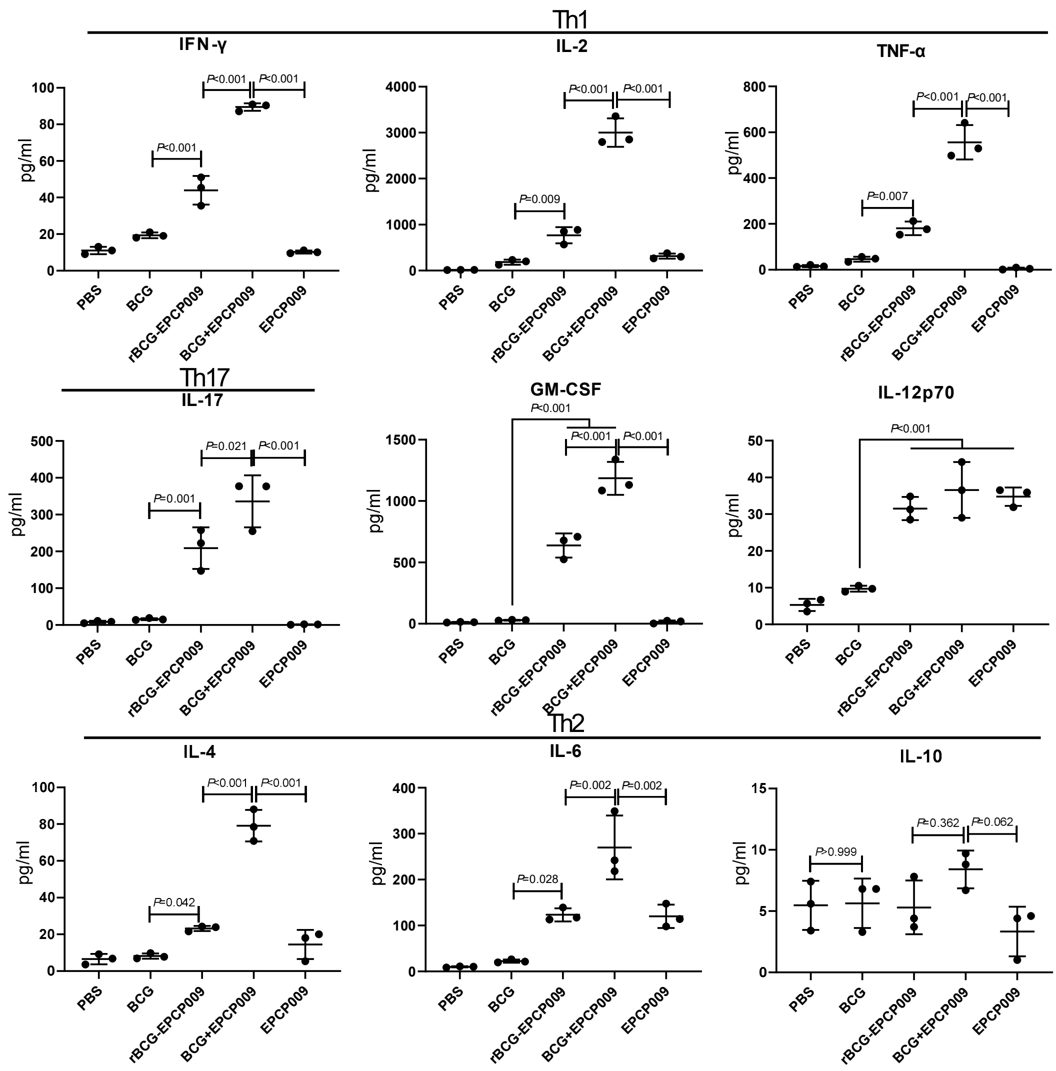

Figure S2. Levels of cytokines secreted by mouse spleen lymphocytes 12 weeks after immuniza-tion with the vaccine candidates. Cytokine levels secreted on stimulation with EPCP009-specific proteins were measured after 12 weeks of initial immunization with the different vaccine candidates. The p-values indicating the significance of differences were determined by one-way ANOVA with Tukey's multiple comparison tests.

Table S1. Nucleotide sequence and amino acid sequence of EPCP009

ATGACAGAGCAGCAGTGGAATTTCGCGGGTATCGAGGCCGCGGCAAGCGCAATCCAGGGAAA
TGTCACGTCCATTCATTCCCTCCTTGACGAGGGGAAGCAGTCCCTGACCAAGCTCGCAGCGGCC
TGGGGCGGTAGCGGTTCGGAGGCGTACCAGGGTGTCCAGCAAAAATGGGACGCCACGGCTAC
CGAGCTGAACAACGCGCTGCAGAACCTGGCGCGGACGATCAGCGAAGCCGGTCAGGCAATGG
CTTCGACCGAAGGCAACGTCACTGGGATGTTCGCAGGTGGTTCTGGCGGTATGAACAATGTGCC
CCAGGCGCTGCAACAGCTGGCCCAGCCCACGCAGGGCACCACGCCTTCTTCCAAGCTGGGTGG
CCTGTGGAAGACGGTCTCGCCGCATCGGTCGCCGATCAGCAACATGGTGTCGATGGCCAACAA
CCACATGTCGATGACCAACTCGGGTGTGTCGATGACCAACACCTTGAGCTCGATGTTGAAGGGC
TTTGCTCCGGCGGCGGCCGCCCAGGCCGTGCAAACCGCGGCGCAAAACGGGGTCCGGGCGAT
GAGCTCGCTGGGCAGCTCGCTGGGTTCTTCGGGTGGTGGTTCTGGCGGTGCAGAGATGAAGAC
CGATGCCGCTACCCTCGCGCAGGAGGCAGGTAATTTCGAGCGGATCTCCGGCGACCTGAAAAC

CCAGATCGACCAGGTGGAGTCGACGGCAGGTTCGTTGCAGGGCCAGTGGCGCGGCGCGGCGG
GGACGGCCGCCCAGGCCGCGGTGGTGCGCTTCCAAGAAGCAGCCAATAAGCAGAAGCAGGAA
CTCGACGAGATCTCGACGAATATTCGTCAGGCCGGCGTCCAATACTCGAGGGCCGACGAGGAG
CAGCAGCAGGCGCTGTCCTCGCAAATGGGCTTCGGTGGTTCTGGCGGTGGTAGCACGCTGCTC
TACCCGCTGTTCAACCTGTGGGGTCCGGCCTTTCACGAGAGGTATCCGAACGTCACGATCACCG
CTCAGGGCACCGGTTCTGGTGCCGGGATCGCGCAGGCCGCCGCCGGGACGGTCAACATTGGG
GCCTCCGACGCCTATCTGTCGGAAGGTGATATGGCCGCGCACAAGGGGCTGATGAACATCGCG
CTAGCCATCTCCGCTCAGCAGGTCAACTACAACAATAGCTCTGGCAATTTCTTGTTGCCCGACG
CGCAAAGCATTCAGGCCGCGGCGGCTGGCTTCGCATCGAAAACCCCGGCGAACCAGGCGATTT
CGATGATCGACGGGCCCGCCCCGGACGGCTACCCGATCATCAACTACGAGTACGCCATCGTCA
ACAACCGGCAAAAGGACGCCGCCACCGCGCAGACCTTGCAGGCATTTCTGCACTGGGCGATCA
CCGACGGCAACAAGGCCTCGTTCCTCGACCAGGTTCATTTCCAGCCGCTGCCGCCCGCGGTGG
TGAAGTTGTCTGACGCGTTGATCGCGACGATTTCCTAG
